# Appearance of post-induction respiratory apnoea in dogs following slow or fast administration of propofol

**DOI:** 10.18849/ve.v11i1.729

**Published:** 2026-01-09

**Authors:** Alexandra Fraser+, Eduardo Uquillas+

**Keywords:** ANAESTHESIA, APNOEA, CANINE, PHARMACOLOGY, PROPOFOL INFUSION RATE, RESPIRATORY DISTRESS

## Abstract

**PICO question:**

In healthy dogs undergoing general anaesthesia is rapid infusion of propofol compared to slow infusion of propofol associated with a greater incidence or duration of post-induction apnoea?

**Clinical bottom line:**

**Category of research:**

Treatment.

**Number and type of study designs reviewed:**

Four prospective, randomised, controlled clinical trials.

**Strength of evidence:**

Weak.

**Outcomes reported:**

The studies have produced inconsistent findings regarding the relationship between propofol infusion speed and post-induction apnoea appearance in dogs. While two studies have found that increasing the speed of administration increases the incidence or duration of post-induction apnoea, other studies have not found a significant correlation.

**Conclusion:**

Based on available evidence, administering propofol at a slow rate is unlikely to lower the incidence or duration of post-induction apnoea compared with faster propofol infusion where the total dose is kept constant. However, administering propofol slowly is recommended when titrating to effect, since slow administration reduces the total dose required to induce anaesthesia, thereby reducing the risk of apnoea.

How to apply this evidence in practice

The application of evidence into practice should take into account multiple factors, not limited to: individual clinical expertise, patient’s circumstances and owners’ values, country, location or clinic where you work, the individual case in front of you, the availability of therapies and resources.

Knowledge Summaries are a resource to help reinforce or inform decision making. They do not override the responsibility or judgement of the practitioner to do what is best for the animal in their care.

## Clinical scenario

You work in a suburban small animal clinic and are planning anaesthesia for a dog with a history of epileptic seizures. You choose propofol as the induction drug but are concerned by the manufacturer’s warning regarding rapid administration and respiratory effects. To address this concern, you decide to investigate the relationship between propofol infusion rate and the occurrence or duration of post-induction apnoea in dogs.

## The evidence

Four randomised controlled clinical trials (Bigby et al., 2017a; Murison, 2001; Raillard et al., 2018; Walters et al., 2022) were found to address whether rapid infusion of propofol, compared with slow infusion of propofol, to induce anaesthesia in healthy dogs was associated with a greater incidence or duration of post-induction apnoea. Each study had methodological limitations and together produced inconsistent findings, providing strong evidence that high doses of propofol increased the incidence and duration of post-induction apnoea, but weak evidence that these findings can be attributed to rapid delivery if dose is kept constant. Overall, the evidence supporting a causative link between propofol administration speed and respiratory apnoea incidence in canines is weak, and further research is required to improve anaesthetic management for canine patients in this regard.

## Summary of the evidence

**Bigby et al. (2017a):** Effect of rate of administration of propofol or alfaxalone on induction dose requirements and occurrence of apnea in dogs **Aim: **To determine how induction dose requirements, in addition to incidence and duration of post-induction apnoea, are influenced by the rate of administration of alfaxalone or propofol in healthy adult dogs premedicated with methadone and dexmedetomidine.

Population:	Healthy client-owned dogs.
Sample size:	32 dogs.
Intervention details:	Study subjects aged between 5 months and 54 months, weighing 25.1 kg ± 23.1 kg, undergoing desexing surgery were included. Brachycephalic breeds, patients with cardiorespiratory compromise, and dogs receiving sedative medication were excluded. Subjects were all premedicated intramuscularly with methadone (0.5 mg/kg) and dexmedetomidine (5 μg/kg). Sedation was subjectively assessed 30 minutes after premedication by a trained evaluator. Subjects were preoxygenated for 5 minutes with a mask attached to a rebreathing system, using oxygen flow of 4 L/min. Subjects were randomly allocated to four groups: Alfaxalone IV 0.5 mg/kg/min (‘A-slow’); n = 8/32 Alfaxalone IV 2 mg/kg/min (‘A-fast’); n = 8/32 Propofol IV 1 mg/kg/min (‘P-slow’); n = 8/32 Propofol IV 4 mg/kg/min (‘P-fast’); n = 8/32. Following preoxygenation, propofol was administered via a syringe driver. A single anaesthetist performed standardised intubation for all subjects. Isoflurane was delivered at 2% in oxygen at a flow rate of 2 L/min using a rebreathing system. All subjects were administered lactated Ringer’s solution (compound sodium lactate) for the duration of anaesthesia intravenously at a rate of 10 mL/kg/hr. During apnoea, only heart rate and oxygen saturation were monitored. Following commencement of spontaneous breathing, or after 3 minutes of intubation, additional monitoring began for blood pressure, carbon dioxide levels, and ECG. Measurements were taken every 5 minutes. Body temperature was measured every 15 minutes using an oesophageal probe. If oxygen saturation fell below 90% or carbon dioxide levels exceeded 60 mmHg after the initial anaesthesia phase, the experiment was stopped and manual ventilation was initiated. MAP readings < 60 mmHg indicate hypotension and were addressed by decreasing the vaporiser settings 0.5% every 5 minutes or by administering a lactated Ringer’s solution fluid bolus (10 mL/kg over 10 minutes) intravenously. Alternatively, dopamine was administered intravenously starting at 7 μg/kg/min and adjusted depending on MAP response. All subjects received 0.2 mg/kg meloxicam at the end of anaesthesia, administered subcutaneously.
Study design:	Prospective, randomised clinical trial.
Outcome Studied:	Incidence and duration of apnoea were recorded and compared across the four intervention groups, where apnoea was defined as cessation of breathing for at least 30 seconds. Apnoea ended when spontaneous breathing resumed. Total induction agent dose was recorded and compared between the four intervention groups. End-tidal partial pressure of carbon dioxide was compared over time across the intervention groups.
Main Findings(relevant to PICO question):	There was a significant difference in induction dose in mg/kg between the P-Slow (n = 8/32) and P-Fast (n = 8/32) groups (P = 0.007). The induction dose was 1.8 ± 0.6 mg/kg for the P-Slow (n = 8/32) group and 4.1 ± 0.7 mg/kg for the P-Fast (n = 8/32) group. There was a significant difference in apnoea incidence between the P-Slow (n = 8/32) and P-Fast (n = 8/32) groups (0% and 100% incidence, respectively) (P = 0.007). Duration of apnoea was significantly lower in the slow propofol administration group (10 ± 8 seconds) than in the fast propofol administration group (247 ± 125 seconds) (P < 0.001). There was a strong positive correlation between increased propofol dose and longer apnoea duration (r = 0.825, P < 0.001).
Limitations:	Anaesthetists were aware of the study protocols, which could introduce measurement and observer bias into the results despite efforts to standardise methodology. This study compared only two propofol administration rates, limiting its ability to determine an optimal induction rate for clinical practice. Range of values for apnoea duration was reported as 10 ± 18 seconds. This is likely an error since negative values for apnoea duration are not feasible.

**Murison (2001):** Effect of propofol at two injection rates or thiopentone on post-intubation apnoea in the dog **Aim: **To quantify and compare the incidence and duration of apnoea following endotracheal intubation in dogs induced with either thiopentone or propofol, and assess the effect of propofol induction speed on respiratory depression.

Population:	Healthy entire and desexed client-owned dogs.
Sample size:	66 dogs.
Intervention details:	Study subjects with a mean age of 3.93 years (SD = 2.87) and mean weight 22.85 kg (SD = 8.50), undergoing various types of surgery were included. Boxers, giant breeds, dogs receiving medications, and dogs < 7.5 kg were excluded. Dogs were premedicated with intramuscular acepromazine (0.05 mg/kg) and morphine (0.25 mg/kg) 30 minutes prior to induction of anaesthesia. Preoxygenation was not performed. Subjects were randomly allocated to three groups: Thiopentone IV 10 mg/kg injected over 2–4 seconds as an active comparator intervention; n = 22/66 Propofol IV 4 mg/kg injected over 2–4 seconds (‘rapid’); n = 22/66 Propofol IV (4 mg/kg) injected over 30 seconds (‘slow’); n = 22/66. Dogs that were insufficiently anaesthetised were removed from the study. A gas mixture of 67% nitrous oxide and 33% oxygen was delivered at a rate of 200 mL/kg/min, with a halothane vapouriser setting of 1.5%. If a dog did not take a spontaneous breath after 60 seconds, a single manual lung inflation was performed.
Study design:	Prospective, non-blinded, randomised, controlled, clinical trial.
Outcome Studied:	Apnoea incidence and duration were recorded, where apnoea was defined as ‘cessation of spontaneous respiration for 15 seconds or longer’. Time to first breath was measured. Respiratory rate and minute volume were measured for the first 5 minutes of anaesthesia.
Main Findings (relevant to PICO question):	There was no significant difference in the incidence of apnoea between the rapid (n = 22/66) and slow (n = 22/66) propofol infusion groups (59% and 64% incidence, respectively). Time to first breath was significantly shorter in the propofol rapid infusion group (median = 19.5 seconds) compared with the propofol slow infusion group (median = 28.8 seconds) (P < 0.05). Respiratory rates differed significantly between the slow and rapid propofol infusion groups during the second, third, and fourth minutes of anaesthesia (P < 0.05). However, this disparity did not impact overall apnoea occurrence. There was no significant difference in minute volume (Minute Ventilation Index, MVI) between the two propofol infusion groups, initially low volume then increasing rapidly during the first 5 minutes of anaesthesia in both groups.
Limitations:	Records of the time elapsed between administration of the induction agent and connection of the breathing system were not provided. This omission is a study limitation as it introduces variability in time-to-first-breath measurements, which only capture the period after breathing system connection and do not consider inconsistencies in the interval between drug administration, intubation, and system connection. Both propofol administration speeds (4 mg/kg over 2–4 seconds and 4 mg/kg over 30 seconds) were relatively rapid compared with those currently recommended by manufacturers (4 mg/kg/min). As such, both groups received a faster-than-recommended infusion, limiting the scope for clinical application of the findings. This study compared only two propofol administration rates, limiting its ability to determine an optimal induction rate for clinical practice. A full dose of propofol, kept consistent for all subjects, was administered without titration. This created a risk of relative propofol overdose, potentially influencing study outcomes by increasing respiratory depression.

**Raillard et al. (2018):** Effect of predosing versus slow administration of propofol on the dose required for anaesthetic induction and on physiologic variables in healthy dogs **Aim: **To evaluate the effects of propofol predosing compared with slow administration on total induction dose and associated cardiorespiratory effects in healthy dogs.

Population:	Healthy client-owned dogs.
Sample size:	32 dogs.
Intervention details:	Study subjects aged between 6–144 months, weighing between 3.5 kg and 47.2 kg, with an ASA score of I or II, undergoing elective surgical procedures were included. Brachycephalic and giant breeds, patients with a high regurgitation risk, nervous or aggressive dogs, patients receiving simultaneous medical treatment, and patients with systemic disease or trauma were excluded. All subjects were premedicated intramuscularly using 0.025 mg/kg acepromazine and 0.25 mg/kg methadone. Subjects were randomly divided into three groups: Propofol predosing: 0.5 mg/kg propofol over 1–3 seconds then, 2 minutes later, 4.0 mg/kg/min propofol; n = 11/31 Control propofol: 0.5 mg/kg saline over 1–3 seconds then, 2 minutes later, 4.0 mg/kg/minute propofol; n = 10/31 Slow injection of propofol: 1.3 mg/kg/min propofol; n = 10/31. An anaesthetist who was aware of study groups set the propofol infusion rate based on group assignment using a syringe driver, which was then covered. A blind anaesthetist was introduced 2 minutes following commencement of the anaesthetic induction protocol to assess adequacy for endotracheal intubation. Anaesthetic depth was continuously evaluated based on muscle tone, palpebral reflex, eye position, and jaw tone. Propofol infusion was stopped immediately prior to endotracheal intubation. Following endotracheal intubation and cuff inflation, subjects were placed in lateral recumbency and connected to a breathing system delivering isoflurane gas in oxygen. Quality of induction and intubation were scored using descriptive scales. Any dogs which became apnoeic within 2 minutes of propofol administration were excluded from the study. If any dogs experienced apnoea for >30 seconds, manual ventilation was performed twice per minute until spontaneous ventilation resumed.
Study design:	Randomised, blinded clinical study.
Outcome Studied:	Apnoea was recorded in patients where apnoea duration exceeded 30 seconds. The total dose of propofol required to allow intubation was recorded. Pulse rate and respiratory rate measured prior to administration of any medications, 30 minutes following premedication, immediately prior to anaesthetic induction (T0), 2 minutes following induction (T2), immediately following intubation (T3), 2 minutes following intubation (P1), and 5 minutes following intubation (P2).
Main Findings(relevant to PICO question):	Propofol dose was significantly lower in the slow propofol group (n = 10/31) (3.7 ± 1.1 mg/kg) compared with the predosed propofol group (n = 11/31) (5.0 ± 1.0 mg/kg; P = 0.002) and control group (n = 10/31) (4.8 ± 0.6 mg/kg; P = 0.012). The difference in apnoea incidence between groups was not statistically significant (P = 0.0340). No significant differences in sedation and activity scores, induction quality scores, pulse rate, respiratory rate, or MAP between groups.
Limitations:	A continuous rapid rate of propofol infusion was not investigated, rather, this study investigated propofol predosing. This study compared only two propofol administration rates, limiting its ability to determine an optimal induction rate for clinical practice. The fastest propofol infusion rate tested was set at the lower end of manufacturer-recommended administration speeds, meaning that the expected contrast in apnoea incidence between a fast and slow propofol administration may not have been observed in this study. Post hoc statistical tests performed by the authors revealed that the study size (n = 32) was insufficient to show significant differences in independent variables between the control and predosed groups, and that 66 dogs per group would have been required to achieve appropriate power.

**Walters et al. (2022):** Determining an optimum propofol infusion rate for induction of anaesthesia in healthy dogs: a randomized clinical trial **Aim: **To elucidate the optimal infusion rate of propofol for induction of anaesthesia in healthy dogs by comparing intubation time and duration of post-induction apnoea between several infusion rates.

Population:	Healthy client-owned dogs.
Sample size:	60 dogs.
Intervention details:	Study subjects with a median age of 22 months (ranging 8 months to 112 months), and median weight of 11.5 kg (ranging 3 kg to 44.3 kg), undergoing desexing or radiographic procedures were included. Brachycephalic breeds, patients with history of regurgitation, and dogs receiving medication were excluded. All subjects were premedicated intramuscularly using 0.5 mg/kg methadone and 5 μg/kg of dexmedetomidine. All subjects were preoxygenated for 5 minutes using an oxygen flow of 4 L/min. Subjects were randomly divided into five groups: Group A: Propofol IV 0.5 mg/kg/min; n = 12/60 Group B: Propofol IV 1.0 mg/kg/min; n = 12/60 Group C: Propofol IV 2.0 mg/kg/min; n = 12/60 Group D: Propofol IV 3.0 mg/kg/min; n = 12/60 Group E: Propofol IV 4.0 mg/kg/min; n = 12/60. An independent observer set the propofol infusion rate based on group assignment and used a concealed syringe driver. Although 6 mg/kg of propofol was drawn-up, the drug was administered to effect, so total induction dose was determined post-intubation. Intubation was performed by a blinded anaesthetist using standardised criteria. Propofol infusion was paused before intubation, resumed for failures, and subjects were excluded after two failed intubation attempts. Subjects were connected to a breathing system delivering oxygen gas at 2 L/min. Following endotracheal tube cuff inflation, no further manipulation was permitted until the first spontaneous breath occurred. Following the first breath, isoflurane was administered via the aforementioned breathing system.If the oxygen saturation of a subject fell below 90%, manual ventilation was performed to a pressure of 12–15 cm H_2_O at four breaths/min until spontaneous breathing commenced or oxygen saturation increased to 95% or higher.
Study design:	Prospective, randomised, blinded clinical trial.
Outcome Studied:	Apnoea duration was measured from intubation to either the start of spontaneous breathing or the initiation of manual ventilation (if required). The total dose of propofol required to allow intubation was recorded. Time points for the following events were recorded by a blinded observer: cessation of propofol infusion, successful intubation, and first spontaneous breath.
Main Findings (relevant to PICO question):	The mean dose of propofol, duration of apnoea, and intubation time were significantly different among groups (P < 0.001, P = 0.017, and P < 0.001, respectively). The total dose of propofol administered to induce anaesthesia was significantly smaller in Group B (n = 12/60) (2.1 ± 0.5 mg/kg) than in Groups C (n = 12/60) (3.4 ± 0.9 mg/kg; P = 0.037), D (n = 12/60) (3.8 ± 0.8 mg/kg; P = 0.003) and E (n = 12/60) (3.9 ± 1.3 mg/kg; P = 0.004). There was no statistically significant difference between total propofol dose administered to Group B (n = 12/60) and Group A (n = 12/60) (1.6 ± 0.8 mg/kg; P = 0.917). koPropofol infusion rate significantly affected apnoea duration (P = 0.004), with a significantly lower adjusted mean duration of apnoea in Groups A (n = 12/60) and B (n = 12/60) (49 ± 39 seconds and 67 ± 37 seconds, respectively) compared with Groups C (n = 12/60), D (n = 12/60), and E (n = 12/60) (207 ± 34 seconds, 192 ± 36 seconds, and 196 ± 34 seconds, respectively) (P < 0.05). Only dogs in Group D (n = 12/60) (3.0 mg/kg/min) required manual ventilation due to desaturation. These three dogs experienced desaturation after 96, 394, and 404 seconds of apnoea, respectively. Intubation time was significantly shorter in Group B (n = 12/60) (115 ± 10 seconds) than in Group A (n = 12/60) (201 ± 10 seconds) (P < 0.0001), with no significant differences in intubation time between Groups C (n = 12/60), D (n = 12/60), and E (n = 12/60). A propofol administration speed of 1.0 mg/kg/min, as used for Group B (n = 12/60), offered the best compromise between speed of induction and duration of postinduction apnoea.
Limitations:	Manually ventilating desaturated patients artificially reduced the recorded apnoea time, affecting data accuracy. Two outliers were identified in the apnoea duration datasets of both Group A and Group B, so were excluded from further statistical analysis. Each intervention was applied to 12 dogs, meaning that approximately 17% each of these two groups was excluded based on an interquartile range method. Data-editing in this way, with such small intervention groups (n = 12), increases the risk of Type I error and artificially identifying a statistical difference.

## Appraisal, application and reflection

Propofol, the most widely used intravenous induction agent in both human and veterinary medical fields, is relatively well understood in both its action and potential to produce adverse effects (Bigby 2018; Smith et al., 1993). Propofol acts on the central nervous system via direct and indirect effects on GABA_A_ receptors to produce either anaesthesia or hypnotic sedation depending on administration protocol, and may also affect the N-methyl-D-aspartate (NMDA) subtype of the glutamate receptor, the inhibition of which further contributes to the ability of propofol to induce anaesthesia (Bigby, 2018; Orser et al., 1995). Propofol has a number of notable advantages over other induction agents including its ability to be administered intravenously without significant cumulative effects upon repeat administration, produce rapid induction and smooth recovery without an excitatory phase, and, importantly, be used in canine and feline patients with pre-existing status epilepticus, hepatic and renal disease (Cochrane, 2007; Glowaski & Wetmore, 1999; Muir & Gadawski, 1998). However, propofol also has a number of limitations, reportedly producing high rates of post-induction apnoea, cardiorespiratory depression, and hypotension (Cattai et al., 2018; Muir & Gadawski, 1998). The clinical implications of prolonged respiratory apnoea for small animal patients involve simultaneous decreases in alveolar gas exchange and gaseous anaesthetic intake, thereby increasing the risk of life-threatening respiratory complications and producing a more challenging environment for safe and effective anaesthetic management of the patient (Keates & Whittem, 2012).

The four studies identified in the literature search strived to identify causative links between rate of propofol administration and the incidence or duration of post-induction apnoea in healthy dogs, and all followed a prospective clinical trial study design. Randomised controlled trials are among the most rigorous study designs, and are particularly valuable when research objectives involve investigating clinical problems pertaining to intervention effects on measurable outcomes, and offering readers applicable solutions to improve patient outcomes (Bhide et al., 2018; Hariton & Locascio, 2018; Sargeant et al., 2014). The Murison (2001), Bigby et al. (2017a), and Raillard (2018) studies may be assigned Evidence Action Ratings (EARs) of B3, B4, and B3 respectively in regard to apnoea incidence if faster propofol infusion is considered as the treatment intervention and slower propofol infusion as standard treatment. Walters et al. (2022) considered propofol duration as an intervention outcome following anaesthetic induction using one of five propofol infusion speeds, making EAR assignment impractical. Very similar study populations were used across the four studies, with brachycephalic breeds and dogs on concurrent medications consistently excluded. However, there are a number of methodological differences between the studies, and each has a number of internal limitations.

Murison (2001) used halothane as the volatile agent during maintenance of anaesthesia whereas Bigby et al. (2017a), Walters et al. (2022), and Raillard et al. (2018) used isoflurane. For measurements of time-to-first-breath these differences will not have impacted results since no gaseous anaesthetic uptake has occurred prior to respiration. However, these differences may contribute to future apnoea phases which occur after breathing circuit connection and the first spontaneous breath, as measured in the Murison (2001), Bigby et al. (2017a), and Raillard et al. (2018) studies. The apnoeic index of halothane is approximately 60% higher than the apnoeic index of isoflurane, meaning that halothane causes greater respiratory depression than isoflurane if given at the same gas concentration (Dunlop, 2014). This difference may have caused a higher overall apnoea incidence in the Murison (2001) study population, since respiratory depression can cause hypoventilation eventually leading to complete cessation of spontaneous respiration (Taenzer & Havidich, 2018).

There is also a possibility that study outcomes were influenced by the choice of premedication drugs in each study. Murison (2001) and Raillard et al. (2018) utilised acepromazine whilst Bigby et al. (2017a) and Walters et al. (2022) used dexmedetomidine in their premedication protocols. Raillard et al. (2018), Bigby et al. (2017a), and Walters et al. (2022) also used methadone. These drugs and drug combinations have been found to have no significant impact on the incidence or duration of apnoea following induction with propofol in dogs (Bigby et al., 2017b; Bigby, 2018). However, Murison (2001) utilised morphine, which is known to cause respiratory depression and upper airway collapse, as a second premedication drug (Freire et al., 2022). Whilst this is possibly a confounding factor in the Murison (2001) study, some research has suggested that morphine is a less potent cardio-pulmonary depressor than methadone in dogs (Maiante et al., 2009). Together, existing evidence suggests that premedication selection is unlikely to have introduced significant variability to findings across the four appraised studies (Bigby et al., 2017a; Murison, 2001; Raillard, 2018; Walters et al., 2022).

Additionally, propofol was administered intravenously using a syringe-driver in the Bigby et al. (2017a), Walters et al. (2022), and Raillard et al. (2018) studies, compared with manual syringe depression by Murison (2001). This means that administration speeds were less consistent within the Murison (2001) study than in the other two papers, potentially introducing variability and creating a comparative limitation. Additionally, both administration speeds used in the Murison (2001) paper are rapid relative to the manufacturer-recommended administration speed, meaning that the study did not investigate the effects of a true ‘slow’ rate, as might be used in clinical practice (Zoetis, 2022). This creates an important limitation to the applicability and reliability of conclusions drawn from the Murison (2001) paper. In the Raillard et al. (2018) study, too, even the fastest propofol administration speed was set at the low end of manufacturer recommended administration rates. This may have resulted in expected differences in apnoea incidence between fast and slow groups not being observed and makes it challenging to compare findings with those reported by Murison (2001).

A further difference between the studies was in relation to the definition of apnoea used. Murison (2001), Bigby et al. (2017a), and Raillard et al. (2018) defined apnoea in terms of time elapsed under anaesthesia with no spontaneous respiration. By contrast, Walters et al. (2022) defined apnoea in terms of time from intubation to commencement of either spontaneous breathing or manual ventilation. These differences in apnoea definition are not study limitations per se, but must be taken into account when comparing study findings and drawing conclusions to improve evidence-based clinical decisions. Finally, Bigby et al. (2017a) and Raillard et al. (2018) both used a sample size of 32 dogs, which is relatively small compared with the Murison (2001) and Walters et al. (2022) studies. In the Bigby et al. (2017a) paper, sample size and power calculations were performed prior to study commencement and statistically significant results were identified, suggesting that sample size is unlikely to have created a true limitation. In the Raillard et al. (2018) study, however, post hoc statistical tests revealed that this sample size has insufficient power to accurately identify significant differences in independent variables between the control and fast administration groups. This may have compromised the accuracy of findings reported by the paper.

The first finding identified by both Bigby et al. (2017a) and Walters et al. (2022) relates to the effect of total propofol dose on the incidence of post-induction apnoea. Though this finding does not directly relate to the PICO question, it is a notable confounding factor when investigating the effects of administration speed for a drug that is commonly titrated to effect in clinical practice. The dose-dependent cardiopulmonary depression caused by propofol has been extensively studied in both veterinary and human medical literature, and there is strong evidence linking high propofol doses with a higher incidence of respiratory apnoea in dogs (Muir & Gadawski, 1998). In a canine dose-escalation study, Keates and Whittem (2012) confirmed the positive correlation between propofol dose and apnoea incidence identified by Bigby et al. (2017a) and Walters et al. (2022), and a human paediatric medicine study has indicated this dose-dependent increase is non-linear, instead involving a plateau or decrease in apnoea incidence for certain dose ranges before the increase continues (Aun et al., 1992). Walters et al. (2022) also discovered that apnoea duration significantly increases with increasing propofol dose, a finding which too reflects existing evidence of this correlation in human anaesthesiology (Park et al., 1997). Bigby et al. (2017a) accounts for differences in apnoea incidence between slow infusion of propofol (P-Slow) and fast infusion of propofol (P-Fast) groups by suggesting that rapid administration of drugs causes accumulation in the plasma before penetrating the central nervous system (CNS), due to a constant equilibration rate of the drug concentration between plasma and CNS. Once the entire induction dose has been transferred to the CNS, the drug is in relative excess, causing increased respiratory and nervous depression and an elevated risk of apnoea (Bigby et al., 2017a). Bigby et al. (2017a) linked this finding to one of the study hypotheses, which was that the induction dose of propofol required to induce anaesthesia is higher when the drug is administered quickly, compared with the required dose when propofol is administered slowly. They concluded that reducing the administration speed of propofol lowers the risk of a relative overdose, thereby minimising apnoea incidence (Bigby et al., 2017a). Similar findings were identified by Raillard et al. (2018), where slow propofol administration was found to reduce induction dose requirements, compared to the other two treatment groups, fast propofol administration, and placebo, respectively. However, unlike Bigby et al. (2017a) and Walters et al. (2022), Raillard et al. (2018) found no significant difference in apnoea incidence between the predosed and slow infusion groups. A similar relationship has also been found between propofol concentration and dose required to induce anaesthesia, where diluted propofol significantly lowers the dose required to induce anaesthesia (Rögels & Martinez-Taboada, 2021). By contrast, Murison (2001) kept the total dose, in mg/kg, of propofol administered to each dog consistent, only varying the speed at which this dose was administered.

A further objective of the studies was to measure the effect that speed of propofol administration has on the generation and appearance of post-induction apnoea, closely reflecting the PICO question. Murison (2001) aimed to quantify the incidence and duration of post-induction apnoea in canine subjects in response to a faster or slower propofol infusion. Their findings suggest that a slower propofol infusion rate is associated with a higher incidence and duration of post-induction apnoea though this difference was not statistically significant (Murison, 2001). Significant differences were found, however, between the slow and fast administration groups in time elapsed before first breath (Murison, 2001). Although time elapsed before first spontaneous breath was not included in the Murison (2001) definition of respiratory apnoea, this metric was included in the Walters et al. (2022) definition. As such, the significant difference in time to first breath identified between fast and slow propofol infusion groups by Murison (2001), with slow propofol administration resulting in a longer time to first breath, directly contradicts the findings of Walters et al. (2022). Importantly, the Murison (2001) study kept the propofol dose, in mg/kg, constant for all subjects. By contrast, in the Bigby et al. (2017a) and Walters et al. (2022) studies, there were significant differences in total induction agent dose between propofol administration groups, as well as a strong positive correlation between propofol dose and both apnoea incidence and duration. Raillard et al. (2018) found no significant difference in apnoea incidence between slow infusion and predosed groups. This makes it challenging to determine whether the effect on the appearance of apnoea in some studies was caused by speed of administration, by dose, or by a combination of both factors. As such, the association between administration speed and apnoea incidence is more clearly characterised in the Murison (2001) study, where administration speed was the only independent variable, than in the Bigby et al. (2017a), Walters et al. (2022), or Raillard et al. (2018) studies where both administration speed and propofol dose varied significantly between intervention groups. Despite this, study outcomes from both Bigby et al. (2017a) and Walters et al. (2022) agree that faster speeds of propofol administration were associated with both significantly higher rates of apnoea incidence, as well as significantly higher propofol doses. Hence, considering the key findings from each of the four studies, current evidence inconsistently characterises the relationship between speed of propofol administration and the appearance of respiratory apnoea in dogs. Cuniberti et al. (2023) compared target-controlled propofol infusion with continuous rate infusion. While the study reported incidental findings regarding administration speed and apnoea incidence, which were consistent with Raillard et al. (2018), it was excluded from this Knowledge Summary as its primary research aims and methodological framework were not aligned with the defined PICO question.

Similarly, Khojasteh & Vesal (2023) found that apnoea incidence was not influenced by a significant difference in propofol infusion rate between two intervention groups, as part of a reflex assessment study in anaesthetised dogs where propofol was utilised for anaesthetic induction and maintenance. As for Cuniberti et al. (2023), Khojasteh & Vesal (2023) was excluded from this Knowledge Summary since their research objectives and study design did not aim to elucidate the effect of propofol infusion speed on apnoea in dogs.

In conclusion, the evidence that a higher propofol dose is associated with greater post-induction apnoea incidence in dogs is strong, supported by findings from two out of four studies, alongside reasonable evidence to suggest that higher doses are also associated with longer apnoea duration (Bigby et al., 2017a; Walters et al., 2022). However, evidence suggesting that slow administration of propofol lowers respiratory apnoea incidence when compared with fast administration of propofol if total propofol dose is kept constant remains weak, since findings from the four examined studies inconsistently supported this (Bigby et al., 2017a; Murison, 2001; Raillard et al., 2018; Walters et al., 2022). It follows, however, from the Bigby et al. (2017a) and Walters et al. (2022) studies, that rapid intravenous administration of propofol during anaesthetic induction when titrating to effect consistently leads to administration of a higher total dose when compared with a slower injection speed, due to both practical limitations of this method – such as waiting to observe clinical effects before ceasing propofol infusion – as well as altered dose requirements depending on administration speed (Raillard et al., 2018). As such, a clinical takeaway from these studies is that administering propofol slowly is likely to minimise effects associated with high doses, including the incidence and duration of apnoea.

Future research may choose to investigate whether similar findings are established if brachycephalic breeds are included in studies of apnoea appearance and the effects of propofol administration speed. At present, no studies have examined whether these breeds demonstrate different outcomes compared with those described by Murison (2001), Bigby et al. (2017a), and Walters et al. (2022), despite having significant anatomical deviations from non-brachycephalic dogs that may exacerbate respiratory complications when under anaesthesia (Gruenheid et al., 2018). Additionally, a small number of studies have examined the relationship between propofol administration methods and intra-anaesthetic hypotension incidence, and this remains an area for further research – in particular, the effect of propofol administration speed on hypotension incidence and severity. Measurement of hypotension, and its occurrence relative to plasma concentration targets during propofol infusion, was one study outcome of the Cuniberti et al. (2023) study. A similar study was conducted by Musk et al. (2005) to analyse anaesthetic induction targets and apnoea incidence in response to increased plasma concentration of propofol. This association also warrants further investigation. Finally, Walters et al. (2022) identified a need for further research to identify propofol bolus administration rates that minimise adverse effects, including apnoea.

## Methodology

**Search Strategy:** 

Databases searched and dates covered:	National Library of Medicine via Pubmed (2000 to Feb 2025) Scopus via ScienceDirect(2000 to Feb 2025) CABI: CAB Abstracts via Web of Science (2000 to Feb 2025)
Search strategy:	For Pubmed, ScienceDirect, and CAB Abstracts:(‘dog’ OR ‘canis lupus familiaris’ OR ‘canine’) AND (‘propofol’ OR ‘propofol induction’) AND (‘apnoea’ OR ‘apnea’ OR ‘respiratory distress’) AND ('rate')
Dates searches performed:	09 February 2025

**Exclusion / Inclusion Criteria:** 

Exclusion:	Publications not relevant to the PICO question, article summaries, literature reviews, case reports, case studies, conference proceedings.
Inclusion:	Publications relevant to the PICO question, randomised controlled trials, clinical trials, randomised crossover study.

**Search Outcome:** 

Database	Number of results	Excluded – literature review	Excluded – article summaries	Excluded – conference proceedings	Excluded – case report/study	Excluded – irrelevant to the PICO	Total relevant papers
PubMed	26	0	0	0	0	22	4
Scopus	15	0	0	0	0	12	3
CAB Abstracts	44	1	0	1	2	36	4
Total relevant papers when duplicates removed							4

## Acknowledgements

Thank you to Dr Eduardo Uquillas, (BVM, DVM, DACVAA) for supervising the preparation of this Knowledge Summary and providing a wealth of experience, knowledge and guidance.

## Author contributions

Alexandra Fraser: Project administration, Conceptualisation, Methodology, Investigation, Writing - Original Draft, Writing - Review and Editing, Visualisation. Eduardo Uquillas: Supervision, Conceptualisation, Methodology, Validation, Writing - Review and Editing.

## ORCiD

Alexandra Fraser: https://orcid.org/0009-0001-9585-0360

Eduardo Uquillas: https://orcid.org/0000-0002-4227-2173

## Conflict of Interest

The author declares no conflicts of interest.
